# Policy learning in the Eurozone crisis: modes, power and functionality

**DOI:** 10.1007/s11077-015-9236-7

**Published:** 2015-12-12

**Authors:** Claire A. Dunlop, Claudio M. Radaelli

**Affiliations:** Department of Politics, University of Exeter, Amory Building, Exeter, EX4 4RJ UK

**Keywords:** Ambiguity, European Semester, European Union (EU), Eurozone crisis, Fiscal policy, Policy learning

## Abstract

In response to the attacks on the sovereign debt of some Eurozone countries, European Union (EU) leaders have created a set of preventive and corrective policy instruments to coordinate macro-economic policies and reforms. In this article, we deal with the European Semester, a cycle of information exchange, monitoring and surveillance. Countries that deviate from the targets are subjected to increasing monitoring and more severe ‘corrective’ interventions, in a pyramid of responsive exchanges between governments and EU institutions. This is supposed to generate coordination and convergence towards balanced economies via mechanisms of learning. But who is learning what? Can the EU learn in the ‘wrong’ mode? We contribute to the literature on theories of the policy process by showing how modes of learning can be operationalized and used in empirical analysis. We use policy learning as theoretical framework to establish empirically the prevalent mode of learning and its implications for both the power of the Commission and the normative question of whether the EU is learning in the ‘correct’ mode.

## Introduction and motivation

Since the early days of policy analysis and organizational theory, there has been substantial academic interest in the concept of policy learning to explain change particularly under conditions of ambiguity (Cyert and March 1963; Lindblom 1959). Indeed, learning seems a suitable way to theorize how constellations of actors manipulate the social construction of policy problems, exploit opportunities for entrepreneurship, and actively use institutions and decision-making.

The European Union (EU) provides a strong most-likely case for the study of collective learning—as defined in Heikkila and Gerlak (2013). This is for two reasons. First, because of its democratic deficit, the EU, especially its bureaucracy, the European Commission, seeks to create legitimacy by drawing on expertise (Verdun 1999; Zito 2001), technical–economic efficiency (Majone 1996) and policy transfer (Zito and Schout 2009). EU policy reforms are often presented in the language of efficiency and learning from ‘best evidence’. But second, and more substantively, EU policy-making is characterized by high levels of ambiguity (Zahariadis 2008). Often EU institutions agree on a decision, but disagree on the implications of the decisions (Howorth 2004). In implementing these decisions, actors may be strategic, but (because of ambiguity) they ‘gamble’ rather than ‘calculating’ (Jabko 2006). Interpretations, ideas and social constructions matter in these contexts of strategic interaction—a territory where theories of policy learning perform well (Freeman 2006; Heikkila and Gerlak 2013).

Since 2008, the sovereign debt crisis has forced the EU to adjust existing policy instruments and design new ones, radically changing how information is generated and acquired for the purposes of monitoring and surveillance. Information acquisition is a fundamental component of collective leaning (Heikkila and Gerlak 2013, see below). The introduction of new information-rich instrumentation draws on existing frameworks, notably the stability and growth pact (SGP) (de la Porte and Heins 2015; Ioannou et al. 2015; Salines et al. 2012). But, *taken together* the information environment and the rules for sanctioning deviant behaviour are qualitatively different from the pre-crisis situation.

For policy-makers, the key question is whether the new governance architecture works or not. Do governments exploit ambiguity, re-negotiating everything that was agreed at the stage of decision-making? Are they converging towards post-austerity economic paradigms instead? Do they change their preferences about budgets and structural reforms? Or, does compliance with the policy instruments for fiscal coordination follow the lines of rule-bound, hierarchical decision-making? All these options make us think in different ways.

We offer a theoretical lens—a framework—to answer these questions empirically. Our framework is policy learning. Doubts and concerns about the values of learning theories loom large (Bennett and Howlett 1992; May 1992). Political scientists agonize about whether the EU learns or not a kind of ‘essentialist’ or ‘ontological question’. They get into troubled waters when they have to explain whether a given episode of change is caused by policy learning and not by another variable—an empirical question. At their roots, these are YES/NO questions. Here, we suggest a different way of looking at policy learning. In doing so, we follow Birchfield (2013): in her analysis of ‘normative power Europe’ she found exactly the same problem of YES/NO questions. To break this impasse, she moves away from these binary questions by taking normative power Europe as framework for the study of the policy process. We do the same for policy learning. We do not try to answer questions about the nature of the EU or the presence–absence of learning. Instead, we start from policy learning as framework and show what can be done with it. Following Birchfield (who draws on Sabatier 2007), a framework for the analysis of the policy process should allow us to elaborate concepts that support conjectures that can be tested empirically. It should provide explanatory leverage. It can also allow researchers to elaborate on the normative implications of their findings (Birchfield 2013: 912).

Our contribution to the literature is theoretical and empirical. Empirically, we show how learning as framework allows us to answer positive questions (and explore their normative implications) about the European Semester, the annual cycle of monitoring and coordination of fiscal policy and structural policy reforms across the EU. Theoretically, our main contribution is to develop conjectures that can be tested empirically (Sabatier’s first criterion for a framework) by adopting a ‘varieties of learning’ framework that up until now has been formulated only in abstract, conceptual terms (Dunlop and Radaelli 2013).

In the section ‘From literature to learning-as-framework’, we illustrate the literature, build our conceptual framework and outline the research design. Then in ‘The European Semester’ introduces information on the European Semester. The section entitled ‘Findings’ is empirical and is dedicated to France and Italy in the annual cycle of EU policy coordination. We use empirical evidence to show how our approach works empirically, concluding that overall the EU is learning in hierarchical and bargaining modes. We then reflect on the implications for the agency of the Commission and power shifts, and conclude with policy recommendations. We finally ask whether these modes are functional—we suggest they are not, because the conditions for their efficient usages are not met (see ‘Discussion and conclusions’).

## From literature to learning-as-framework

There has been fast growth in the academic literature on the responses to the economic and political crisis of the EU (Ioannou et al. 2015; Tosun et al. 2014), but so far there are no studies tackling ambiguity upfront, and no attempt to use policy learning explicitly. This is odd because—as noted by Heikkila and Gerlak (2013)—crises are often exogenous triggers of learning processes and the EU is no exception (Tosun et al. 2014).

Extant literature examines: the causes and the unfolding of the international financial crisis across the EU and its member states (Hardie and Howarth 2013); the regulatory response to the financial crisis in the EU and its member states (Moschella and Tsingou 2013; Quaglia 2012); the building up of the sovereign debt crisis in the Eurozone (Dyson 2014; Tsoukalis 2011) and its cross-country effects (van Hooren et al. 2014); instrument change such as the fiscal compact (Chang 2013), the European Stability Mechanism and its forerunner (Gocaj and Meunier 2013); governance issues (Tosun et al. 2014), institutional change such as the role played by the European Central Bank (Salines et al. 2012) and the European Commission (Schwarzer 2012); inter-governmental deliberation (Puetter 2012; Schimmelfennig 2015); and finally, the social dimension of the crisis (Copeland 2015).

In terms of explicit policy theories, there is a multiple streams analysis of the changing vision for policy reforms in the EU (Copeland and James 2014). Others have drawn on different theoretical frameworks, such as principal-agent analysis (Savage and Verdun 2015) and European integration theories (Ioannou et al. 2015; Schimmelfennig 2014, 2015; Vilpišauskas 2013), without getting into policy theories explicitly. A bit closer to learning theories are those who have addressed the ideational aspects (Blyth 2013) and the narrative dimension of austerity (Schmidt 2013). But, policy learning as theoretical lens remains uncharted territory in this field of studies on the EU and the ‘crisis’—apart from some observations in Neimann and Ioannou (2015).

If, instead, we pan out to the more general literature in EU policy studies, we find that ‘learning instructions’ or ‘learning from the top’ are used frequently to explain how targets and indicators impact on domestic policy choice via processes of decoding, interpretation, editing and creative manipulation (Radaelli 2008). Added to that, learning underpins several theories of policy process. The question then is why is learning rarely used ‘on its own’? We reason that learning is *more than* a component of existing theories of the policy process. It can be a proper framework for the study of the policy process. The challenge is to develop concepts and identify theoretical leverage, test conjectures empirically and, possibly, explore the normative implications of the findings with the aid of the framework. We follow Heikkila and Gerlak (2013) who distinguish between actors (in our case a government, or a Prime Minister) and the system (learning in the European Semester); between the process of learning and the products of learning; and, within learning processes, between acquisition of information, translation and dissemination. We are eminently interested in the *process* of learning, because, once we have adopted learning as framework (as opposed to learning as an essentialist YES/NO question), the key question is whether we can characterize and identify the process of learning. For us, this means that empirically we set out to establish whether there is a prevalent mode of learning in the European Semester, or more specifically in the episodes and time period we examine. This is different from an analysis of the products of learning. To illustrate, one can show that the dominant mode of learning is, for example, reflexivity, but whether this also leads to all or some governments of the EU to learn how to converge on fiscal and structural policies is another, different empirical question—a question that will be answered in the future, when the EU reaches its own self-imposed deadlines for convergence (the year 2020).

Turning to the development of concepts within a framework, what modes of learning do we expect to observe, then? Dunlop and Radaelli (2013) generate four learning types by using two variables. One is the social certification of actors: Is there a socially certified actor that stands out from the crowd in a given social interaction? One can think of organizations like the European Central Bank or the epistemic community of austerity economics (Blyth 2013). The other variable is problem tractability—some policy problems are ‘wicked’ (Rittel and Webber 1973), others have their own stable technologies, a few even have algorithms leading to predictable solutions. Problem tractability under conditions of ambiguity is, at least in part, socially constructed. It is not an absolute given, as demonstrated by the hot debates on whether ‘social investment’ is a solution to the problem of growth or it aggravates budgetary problems.^1^

By combining the two variables, Dunlop and Radaelli (2013) generate four varieties or modes of learning. They are reflexivity, (pluralistic) bargaining, epistemic and hierarchical learning. Learning as social process can either take place in a policy sub-system or organization where an actor has a socially privileged position in relation to knowledge—imagine a ‘teacher’, an ‘expert’, a central bank (epistemic), but also a Court or the European Commission (hierarchical). Or, at the opposite, learning can occur in a constellation of actors that are somewhat equal in relation to how society values their knowledge. This is true of situations of reflexivity and bargaining, when every actor in a given constellation has to accept that any other actor may come up with ‘the best argument’ (reflexivity) or there is a pluralist setting with no monopolistic position (bargaining).

As for problem tractability in our empirical context, we observe variation from total lack of ‘know how’ (in the hardest days of speculative attacks on sovereign debt) to the emergence of instruments of monitoring and fiscal surveillance. When these instruments are used, actors still argue over the meaning of some aspects of the deficit procedures and how the European Commission and the EU Ministers interpret them. Some think that an excessive deficit triggers a ‘flow-chart’: ‘if you do this, we will not let you do that’. Yet, others think the deficit procedure opens room for negotiation, dialogue, perhaps even reflexivity: ‘if you do this, we want to talk to you about that by using our power of surveillance’.

Our inquiry is designed to explore the relationship between the annual cycle of policy coordination and learning, and to answer a question about the dominant learning type(s) in the European Semester. This is our research question. But, the answer to this question will also allow us to draw implications about the agenda-setting power of different actors, specifically the Commission and therefore link our analysis to more ambitious questions about power. Of course, a power analysis should take the position of EU institutions and member states pre-crisis, and examine how power has changed with the crisis, attributing these changes to learning or other variables. Hence we can only dig on the implications of our analysis, instead of providing a power-centred analysis. We will also draw on the findings to investigate the normative dimension of learning-as-framework. To achieve this, we will elaborate in the conclusions of whether the prevalent mode of learning is functional for the EU as complex organization, or the EU is learning in the wrong mode so to speak.

For our empirical analysis, we need expectations. Following Dunlop and Radaelli (2013, pp. 603–604) and Heikkila and Gerlak’s threefold categorization (acquisition, translation and dissemination), we reason that collective learning in the European Semester can occur in the following varieties or modes:

- *Hierarchically*: in a hierarchical mode, we expect the EU institutions to use policy instruments as set of rules, and steer the process from the ‘top’ in the most predictable ways, minimizing negotiations and exceptions to the rules. Learning is about gathering information centrally and translating and disseminating it via instructions supported by incentives and sanctions.
- As by-product of *bargaining*: while learning through bargaining may appear counter-intuitive, negotiation can generate information and update thinking in ways that would not have happened in its absence. But, we expect negotiation to dominate all interactions: pretty much anything can be negotiated. For example, the meanings of reporting and information gathering for concrete reforms (that is, translation) arise out of processes of exchanges of resources. Dissemination hinges on partisan mutual adjustment.
- *Reflexively*, we expect the process of information and knowledge gathering to tap into the benefits of local innovation and diffuse good practice across the system. The key decision-making fora—we expect—will be in search mode, looking for solutions to fiscal problems, unemployment and poverty that originate somewhere in the system, often at the local level (Sabel and Zeitlin 2008). Translation involves changes in the preferences of collective actors (i.e. governments) as a result of communication.
- *Epistemically*, we expect communities of authoritative experts with shared causal beliefs to be key to gathering knowledge, experience and provide conceptual frameworks for ‘translation’ (Haas 1992). Dissemination (of experience of what works) is based on knowledge mechanisms, not on the formal hierarchical powers of the organization. We therefore expect the decision-making fora of the Semester to be open to technocratic solutions and inter-governmental relations to be informed by expert’s advice.

Turning to the normative dimension of learning-as-framework, there are scope conditions for each mode. For hierarchy to be functional, the solution must be known ex ante to reduce ambiguity about the type of information, knowledge and experience that is pushed down the chain of hierarchy. Further, hierarchical learning is not efficient if the principal does not know the production function of the agent. The European Semester indeed has several reporting requirements that should reduce information asymmetry between the Commission and the Council (on one hand) and the member states, on the other. The Commission can also intervene with enhanced monitoring when more information is needed from a country that deviates from fiscal targets. Another condition is that the principal has legitimacy and can deploy sanctions if necessary. If, for example, a Prime Minister complains that Germany is using the European Semester to impose its preferences on the fiscal policy of other member states, then legitimacy is contested and the EU institutions find it harder to deploy the corrective measures of the European Semester. In such circumstances, learning may be blocked as defeatism takes hold.

Learning through bargaining is dominated by the logic of exchange and negotiation. An important condition for efficient pluralism is that conflicts do not have a cumulative nature (Lijphart 1999). Thus, our condition for functional learning under bargaining is that actors play the European Semester negotiations without knowing whether they will gain or lose systematically.

In the reflexivity mode, learning is functional so long as the conditions for communicative rationality and deliberation are met (Risse 2013). There is no assumption that someone has ‘the solution’ or that correct information, knowledge and experience exist ex ante (Sabel and Zeitlin 2008). All this can only be discovered within the network of actors, often exploiting local innovations. If the officers of the European Commission interpret their role in the European Semester as facilitators of dialogue (as opposed to the role of ‘flow-chart’ pilots), then reflexivity is functional. However, if paradigmatic positions are essentially incommensurable, reflexivity is dysfunctional (Pellizzoni 2001). For example, the crisis may require discontinuity and conflict—reflexivity may protect the authority of current decision-makers, especially if deliberation occurs only within small technocratic groups rendering capacity uneven (Joerges and Neyer 1997).

Finally, epistemic learning is functional where the conditions for evidence-based policymaking are met and decision-makers instructed in ‘what works’ (Dunlop and Radaelli 2013). Thinking about our empirical case, the success of this mode is contingent on the presence of a community of experts with shared causal beliefs about cause–effect relationships about the sovereign debt crisis and growth. Empirically we should observe policy sectors of the European Semester (fiscal policy, employment, but also sustainability and social inclusion) conforming to the logic of discovery (as opposed to adversarial power arenas), where each innovation or idea is tested by using evidence pragmatically. But, broadly speaking, the ‘solution’ (such as ‘austerity’ or ‘social investment’ and so on) and the ‘lesson taught’ by the experts to policy-makers must work and in this sense be ‘correct’. As shown by Hirschman (1970), paradigms provide their own blinkers and hindrances to learning when thinking outside the box is vital. Where thinking becomes closed, the potential for groupthink pathologies grows. Innovation is stifled and warning signs ignored that the paradigm is not working according to expectations.

Turning to research design, we seek to identify the predominant modes of collective learning, how functional they are and their power implications by studying one cycle (2013–2014) of the European Semester, although in our project we collated material from official documents since the inception of the Semester. We compiled a chronology of events to support our process tracing and track episode-by-episode the annual cycle, which starts in Fall with the Annual Growth Survey prepared by the Commission and prescribes a certain pattern of reporting and decisions by member states and the EU institutions. We assembled some 250 official documents and gathered additional material from the press and research institutes. Given the obvious limits and length of a single paper, here we focus on the 2013–2014 cycle of the European Semester. This is the fourth cycle where the Semester has become established and when two of the largest countries, France and Italy, entered the grey area of deviations from the convergence targets that can be considered temporary by some and permanent by others. We supplement official sources with material from the Italian and French press.

## The European Semester

Before we appraise the modes one-by-one, we need to provide information on context and empirical settings. In terms of macro-economic policy, the convergence goals are based on economic indicators, but the European Commission also monitors progress towards the wider ‘Europe 2020’ indicators^2^ and social investment targets (European Commission 2013). Thus, the Commission can monitor progress towards fiscal convergence, or lack thereof, by considering a wide range of policy areas, from climate change to social inclusion.

The SGP was reinforced during the crisis with the aim of strengthening preventive and corrective measures [the excessive deficit procedure (EDP)]. The new system of coordination is made up of five regulations and one directive (the so-called six-pack, December 2011) that guide governments towards structural budget balance around their medium-term objectives. The structural budget balance operates as safety margin against breaching the 3 % headline budget deficit threshold of the SGP; it is not sensitive to cyclical changes and one-off measures and for this reason is called ‘structural’. The six-pack contains a macro-economic imbalance procedure (MIP) with 11 indicators—increasing the Commission’s surveillance capability. The six-pack applies to all member states, with some provisions that apply only to the 18 countries of the Eurozone currency area.^3^

The European Semester, introduced in 2010, is a policy-making calendar (summarised in Table 1). Basically, it is an annual cycle of coordination where the governments and the EU institutions discuss their budgets and policy reforms at specific times during the year. Before the crisis, fiscal policy and structural reforms were channelled through different, scattered processes.

**Table 1:** European Semester overview

	European Commission	European Council	Member states	European Parliament
November	Commission publishes annual growth survey (AGS) and alert mechanism report (AMR) Commission opinion on draft budgetary plans of Eurozone countries	Finance ministers discuss Commission opinions on draft budgetary plans		
December	Bilateral meetings with member states		Member states adopt budgets	
January	Fact-finding missions in member states	National ministers adopt conclusions on AGS and AMR EU leaders agree main areas for coordination based on AGS and AMR		
February	Country report per member states (reform agenda and imbalances)			
March	Bilateral meeting with member states	EU leaders adopt economic priorities based on AGS		Dialogue on economic priorities
April			Member states present their national reform programmes (NRPS) (on economic policies) and stability or convergence programmes (on budgetary policies)	
May	Commission proposes country-specific recommendations (CSRs) for budgetary, economic and social policies			
June		National ministers discuss the CSRs		
July		Council adopts final CSRs		
August–September				
October			Member states present draft budgetary plans and economic partnership programmes (EDP countries)	

*Source*: Based on http://ec.europa.eu/economy_finance/economic_governance/the_european_semester/index_en.htm

In the European Semester, the Commission produces the annual growth strategy (AGS, in November), reviews the fiscal and structural reforms of all 28 EU member states (Country Reports in February), provides recommendations (so-called Country-Specific Recommendations in May) and monitors the implementation of the recommendations. The alert mechanism reports (AMRs) provide early warning of potentially harmful deviations from macro-economic balance. The AMRs lead to more monitoring via ‘in-depth reviews’. In the first annual cycle (2010–2011), 12 member states fell into the AMR mechanism, 14 during the following year.

In the second phase of the annual cycle (the National Semester), the governments are supposed to implement the policies they have agreed. This is a system of routinized monitoring, and iterative implementation of reforms. The Commission monitors fiscal coordination before decisions are made at the national level. But it also transforms information gathering into ‘translation’—to use Heikkila and Gerlak’s concept. In fact, it makes recommendations and monitors the performance of individual member states towards the jobs, education, innovation, climate change and poverty reduction targets in the EU’s long-term strategy called Europe 2020 (on the latter, see Copeland and James 2014). Hence, the annual cycle has the potential to cover a broad palette of policy reforms. In terms of ‘dissemination’ (Heikkila and Gerlak’s third dimension of collective learning processes), Eurozone countries are obliged to implement the recommendations of the Commission, unless a qualified majority of those countries (excepting the country in question) is opposed to the decision proposed or recommended. The latter is a provision of the fiscal compact, which we illustrate next.

The fiscal compact, signed in March 2012 (binding for the euro area; other countries can join it entirely or selectively), adds on to the architecture of surveillance and enforcement and is intended to strengthen the SGP—formally, it is Title III of the Treaty on Stability, Coordination and Governance (TSCG).^4^ The assistance provided by the European Stability Mechanism is conditional on the signature of the Treaty and incorporation of the Treaty rules into domestic law, preferably constitutional (art. 3.2 TSCG). The major rules are that the budgetary position of a country should be balanced or in surplus, that the structural deficit should not exceed 0.5 % of the GDP and that the reduction of debts over 60 % of the GDP should take place at one twentieth per year. Art. 3 TSCG stipulates that convergence towards the medium-term objective of a country should be ‘rapid’. Crucially for our analysis of learning, ‘the timeframe for such convergence will be proposed by the European Commission taking into consideration country-specific sustainability risks’. Deviations from the medium-term objective or from the adjustment path are possible only under ‘exceptional circumstances’ (art. 3, par. 1/c and par. 3/b TSCG)—events that are outside the control of the government, ‘provided that the temporary deviation of the Contracting Party concerned does not endanger fiscal sustainability in the medium-term’. Obviously, establishing if a circumstance is exceptional is not automatic, quite the opposite, there are margins of judgement and appreciation. Note that governments have to report both on deficit and on debt. On the latter, the fiscal compact rule is that they have to report ex ante on their public debt issuance plans both to the Council and the European Commission. One important surveillance property of the fiscal compact, indeed, is that governments are obliged to report *exante* on all major policy reforms to the Commission and the Council.

When a government’s economic policy breaches the thresholds of deficit or public debt, the corrective arm enters the scene. The correction mechanism is intrusive in terms of national policy autonomy, since it revolves around ‘common principles to be proposed by the European Commission, concerning in particular the nature, size and timeframe of the corrective action to be undertaken, also in the case of exceptional circumstances, and the role and independence of the institutions responsible at the national level for monitoring compliance’ (art. 3, par. 2 TSCG). The treaty-based EDP gives the Commission a platform to discuss the timeframe, rhythm and path to reforms with the governments that exceed the thresholds. Governments must present a ‘budgetary and economic partnership programme’ that includes ‘a detailed description of the structural reforms which must be put in place and implemented to ensure an effective and durable correction of its excessive deficit’ (art. 5 TSCG).

After surveillance and monitoring, the third component of the mechanism is sanctioning. Deviations from the fiscal compact allow the Commission (or any other government, independently of the Commission’s views) to bring a non-compliant member state to the Court of Justice. If this member state does not subsequently comply with the judgement of the Court, it can be fined up to 0.1 % of GDP.

To recap on the policy coordination cycle (Table 1): after the Annual Growth Survey in November, at the end of the calendar year member states adopt their final budgets taking into account the opinions of the Finance Ministers meeting in the ECOFIN Council and the advice of the Commission. In March, the AGS is discussed by the Parliament and the relevant Council formations. At this time, countries that have been targeted by an AMR because of their imbalances receive the in-depth reviews of the Commission. In April, all countries (first the Eurozone countries and then the others) submit their medium-term ‘stability or convergence programmes’ and ‘national reform programmes’ (NRPs) outlining medium-terms objectives and the policy reforms. In May/early June, the Commission produces its evaluation of the reform plans and issues CSRs. Always in June, the discussion of the CSRs made by the Commission goes up to the Employment Ministers first, then to the Finance Ministers (the ECOFIN Council) and finally to the Council of Prime Ministers and Heads of State (Council of the EU). In July, the Council of the EU releases the final recommendations agreed/formally they are adopted by the ECOFIN Council. The member states submit draft budget plans for the next year by mid-October, and the cycle restarts.

## Findings

In this section, we draw on empirical evidence to appraise the four varieties of learning. Specifically, does evidence allow us to make the case for hierarchy, bargaining, reflexivity or epistemic learning?

### Learning in the shadow of hierarchy

The first, perhaps banal but still important, observation we make is that the rules are enshrined in the Treaty and have legal binding force. Up until now (Fall 2015), no major politically motivated upheaval of the rules of fiscal discipline and the European Semester processes of coordination has occurred. In some episodes, compliance with the rules and processes has come at political costs.

Take the case of Prime Minister Manuel Valls in France in 2014: he did not repeat the move of the French and German governments of the past to use the Council to overturn a difficult relationship with the Commission on the state of play with fiscal discipline (Heipertz and Verdun 2010). The budgetary correction imposed by Valls to the French parliament in the first part of 2014 generated major discontent within the majority and the French Socialist Party—put differently, the measures were politically and electorally painful, but were nonetheless pushed through the Assemblée Nationale.

Second, the pyramid of monitoring, alerts and enhanced surveillance is firmly in place—it is too costly to openly challenge this architecture in the name of ‘post-austerity’ and ‘more growth’. There have been attempts to increase flexibility with ‘social investment’ (see footnote 1), but no one wants to demolish this bastion of credibility *vis à vis* the financial markets. Third, the Commission as custodian of the hierarchical rules has the prime mover’s advantage, with the annual growth survey in November. It can put a government on the spot and draw attention to imbalances that are difficult to ignore. With the two-pack (in force since May 2013), Eurozone countries send their budgetary plans to the Commission by mid-October, allowing a systematic and coordinated scrutiny of their fiscal policies.

The calendar of the European Semester has been respected. Information has been sent out regularly, procedures have been activated, and the preparation of national reforms plans has gained prominence on the domestic policy agendas. ‘What the Commission may say about this delay with the deficit adjustment plan’ and ‘what the Council will say about us’ are topics that are aired every month in the press and parliamentary debates we examined for France and Italy. The stock exchanges in France and Italy reacted quickly to the publication of the Commission’s assessment of national reform plans in June 2014. This contrasts with the low political and media saliency of the reporting steps in the processes of facilitated coordination used in the past. The rules have been hardened (Borrás and Radaelli 2014).

### Learning as bargaining

Let us turn to bargaining now. The key issue in the period we examined was always the same: How much is a ‘given’ and how much can be negotiated? In other words, all governments obviously want to bargain, but what are the boundaries of the negotiable? Let us look at this episode: in September 2014, the Italian Prime Minister Matteo Renzi said to the press that ‘Italy does not take lessons from Europe’—a statement that the new Commissioner for economic and monetary affairs and the Euro, Jyrki Katainen, countered with these words: ‘we are not teachers, we are interpreters’^5^ showing that powerful actors acknowledge there is always room for negotiations.

Fiscal surveillance is definitively not an automatic flow-chart process; it is negotiated. The Commission has agency but under conditions of ambiguity. It can, and indeed does, negotiate. However, the agency of supranational institutions is limited by another important bargaining force at work: the reputational power of Prime Ministers. The Commission and the European Central Bank were hard and uncompromising during the most ferocious speculative attacks on the euro, especially with an executive that was losing prestige and popularity like the late Berlusconi government in Italy.^6^ But, the supranational voice has been less authoritative towards new Prime Ministers with fresh reputational capital, like Valls and Renzi. In turn, these two Prime Ministers have used high politics to try to set limits to the autonomy of the Commission and in attempts to secure favourable treatment from the Council.

France mobilized different high-level government officials to put pressure on the European Central Bank to weaken the Euro in May 2014, thus giving more chances to French competitiveness.^7^ The same officers put pressure on colleagues in other members of the euro area to raise the issue of the Euro exchange rate formally. Recall, that this happened at a time when Valls was pushing through the National Assembly a severe budgetary plan that cost the French Socialist Party several losses in terms of support in Parliament and at the European Parliamentary elections of May 2014.

On 2 June 2014, the Commission evaluated the French national reform plan with little enthusiasm, making a number of specific critical recommendations (European Commission 2014a). As explained above, the recommendations are then discussed in the Council of Ministers and the Council of the EU in June before they are finalized in July. Unsurprisingly, Valls started sending signals to the press that he was negotiating with colleagues in the Council a flexible approach to deficit rules under a period of slow growth in some countries and recession in others, and followed with a more confrontational posture in October 2014. France pushed the boundaries of top-down compliance towards bargaining, if not outright conflict, with Germany and the Commission during that month, when it decided not to go for additional budgetary corrections, thus staying above 3 % until 2017 and risking a fine up to 0.2 % of the GDP (the EDP for France is supposed to be closed with the 3 % threshold reached in 2015). Speaking in London in early October 2014, Renzi said that Italy ran no risk of getting into the EDP but nevertheless was supporting the principles invoked by France: ‘I respect the decision of a free and sovereign country like France. Nobody can treat the other member states the same way one treats students. I stand by [President] Hollande’.^8^

Renzi coordinated this posture with the then Italian President Giorgio Napolitano to create room for bargaining between Italy and the newly appointed Commission: in Fall 2014 the Italians felt that they could not reach the budget target before 2017. In consequence, Renzi, his finance and economy minister Padoan and President Napolitano sought on one hand to support the right of member states to make their voice heard by the Commission, and on the other to exchange reforms like the Italian Jobs Act against flexibility in the interpretation of the fiscal rules—i.e. using the investment lever to counter the economic slowdown. Renzi in several interviews with the press in September and October 2014 used the word ‘exchange’, especially in expressions like ‘our battle for flexibility will allow us to stay for 3 years within the conditions we set [that is, reaching the targets in 2017, Authors’ note] and to exchange flexibility with reforms, facilitating the European investment plan of 300 billion Euro’.^9^

This bargaining logic dates back to the days of Renzi’s appointment on 22 February 2014. Already in Spring, his technical officers and Finance Minister Padoan used a meeting in Washington to sound the then Commissioner Siim Kallas on the likelihood of the Commission accepting a temporary deviation from the convergence target. On 16 April 2014, Padoan wrote a letter to Kallas with the Stability Programme and the National Reform Programme for Italy. In slightly surreal prose, the letter explains the ‘slowdown of the convergence’ in 2014, followed by an expected ‘strong convergence’ in 2015, and ‘full convergence’ in 2016, together with a promise to privatize public assets in the range of 0.7 % of the GPD between 2014 and 2017. Kallas replied on the same day,^10^ saying he was ‘taking note’—a factual point that was interpreted by the Italian press as signal that the Commission was open to negotiations on an Italian slow down of deficit reduction plans.^11^ As mentioned, in Fall 2014 Renzi tried to shift ‘full convergence’ to 2017 instead of 2016.

Renzi has made every possible effort to bargain in the European Semester, exemplified in his statement that governments are not students and the Commission and Germany cannot teach lessons. One bargaining chip that was not played, or arguably was played wrongly, was the Italian Presidency of the EU: Renzi insisted on using the leverage of the Presidency to press on the appointment of Mogherini as the EU’s foreign policy representative. He was successful in that, she was appointed, but this narrowed the opportunities for the Presidency to promote the much-sought (by the Italians) EU-debate on post-austerity economic policy ideas and above all flexibility in the rules.^12^

To conclude, how did France and Italy use the bargaining mode? In February 2015, the Commission gave France a new deadline, granting Paris until 2017 to bring its deficit below the EU limit of 3 %. Renzi cashed on his policy reforms^13^ because the Commission decided not to open an EDP. Reportedly, within the Commission, Moscovici pushed for a more generous deadline for France (2018) but Vice-President Dombrovskis insisted on 2017. Since the French extension materialized in the same days of a very hard re-negotiation of the financial deal with Greece, this looked too much like flexibility *à la carte*^14^ showing that too much bargaining can break down the credibility of the new governance architecture.

### Learning through reflexivity

By contrast, we found only limited evidence for reflexivity. Perhaps the strongest argument for this mode comes from the discussion on social investment within the Employment Council and more generally the European Semester (de la Porte 2014; Zeitlin and Vanhercke 2014). As mentioned, the main thrust of the debate is to consider elements of public expenditure concerning the ‘social dimension’ as investment, thus changing the total figures used to compute deficits. This can be seen as reflexivity in that the European Semester was created to generate macro-economic coordination via surveillance. But (this is the argument) EU policy-makers have now learned that only the ‘social’ can fix the ‘economic’ dimension and deliver on sustainable growth. There are also ideas within the European Commission and certain governments to present a social imbalance procedure to fend off the socially undesirable effects of the macro-economic imbalance procedure^15^ (de la Porte and Heins 2015 suggest something like that when they argue that the Eurozone criteria should be complemented by social investment criteria).

Additional evidence for reflexivity comes from the Commission’s reaction, in June 2014, to the Italian national reform plan. The Commission issued eight recommendations (technically these are ‘recommendations for a Council recommendation’) to the Italian government. The fifth, on labour market reforms, contains a reference, in line with the Council’s endorsement of the social investment strategy (European Council 2013) ‘to address exposure to poverty and social exclusion, scale-up the pilot social assistance scheme, in a fiscally neutral way’ and to ‘improve the effectiveness of family support schemes and quality services favouring low-income households with children’ (European Commission 2014b, p. 7). The Commission pushed on social investment recommendations across the board, especially with Bulgaria, Croatia, Ireland, Lithuania, Poland, Portugal and Romania in the June 2014 country-specific recommendations. However, the operational and budgetary meanings of social investment are contested (Kvist 2015; de la Porte and Heins 2015; Morel et al. 2014). Care institutions and schools are certainly a component of social investment, but do we mean ‘any care’ and ‘any type of school’ or high-quality care and schools?

The ‘social’ remains subordinate to the ‘economic’—the achievement of social investment objectives is still based on voluntary coordination among member states and weak surveillance and enforcement (de la Porte and Heins 2015). The discussion on the social investment dimension has been more enthusiastic in the Council Committees working on employment and social inclusion that in the pivotal ECOFIN Council. The January 2015 communication of the Commission on flexibility (European Commission 2015) essentially restates that the only flexibility allowed to Member States is the one already contemplated in the Treaties and the legal framework for policy coordination. At the Franco-Italian summit in February 2015, Renzi and Hollande argued in press interviews about a change of direction of austerity and flexibility,^16^ but in their official conclusions they had to admit that flexibility is only ‘the one enshrined in the rules’.^17^

### Epistemic learning

Finally, turning to epistemic learning, there is impatience with austerity in academic and policy circles. Books and pamphlets on ‘new models of growth’ by academics and political consultants are now common (e.g. Aghion et al. 2014 on the French debate). There is no doubt that, in the world of ideas at least, though not in the world of public policy, we live in a post-austerity debate. However, an alternative, internally coherent, widely shared (among experts) paradigm has not emerged. There are many possible alternatives: economists and social scientists have not converged on the post-austerity model.

The Commission has mobilized its Horizon 2020 funds for EU-wide research to generate more expertise, with four calls in 2014 for projects dedicated to the crisis. Interestingly, in Horizon 2020 there is an explicit reference to more research on the ideational roots of the crisis, showing that the officers at the Commission believe that policy ideas matter. But ultimately, the Commission has a process goal in ideational politics, not an outcome goal. Given its limited legitimacy and capacity, it can only orchestrate existing research and keep momentum with the flow of ideas. It cannot possibly steer an epistemic community in the field of macro-economic policy and growth and generate consensus towards one model or another.

## Discussion and conclusions

Our evidence is preliminary and limited to two countries and a single cycle of the European Semester. With these caveats, our analysis exemplifies how learning-as-framework performs. Our evidence points to bargaining and hierarchy rather than epistemic learning or reflexivity. As for the second question on the power of institutional actors, the Commission has constrained agenda-setting abilities. Its agency is bounded by high politics, the reputational capital of Prime Ministers and ultimately the agreements taken in the various Council formations. Much of what the Commission does, or does not do, depends on a mechanism of anticipated reactions: we have seen above that technically the Commission presents ‘recommendations for Council’s recommendations’ and there is no point in proposing something aggressive towards a member state if the Council is bound to reject the proposal a few weeks later. Our conclusions support the findings of other recent studies that describe a *redirection* of the roles of the Commission rather than fundamental change and an increase in its implementation competencies—but not necessarily in agenda setting (Savage and Verdun 2015; Schwarzer 2012; Bauer and Becker 2014).

Thus, we can exploit the theoretical leverage of learning-as-framework to build bridges between learning and power. In our cases, to learn is a way to define power relations more precisely, to pin down the area for agency and to test power in crucial episodes. There is another, perhaps more subtle, lesson about power and learning: *actors compete over the selection of learning modes*. The Commission goes for bargaining when this increases its leverage over solutions, but it goes back to the hierarchical rulebook when its power is threatened. Brussels seems to prefer a bargaining mode with France, but can be more hierarchical towards Greece. Future research should establish whether the Commission and the Council select one mode or another depending on the characteristics of a member state.

Finally, this leads us to the normative implications. Bargaining and hierarchy prevail, but the conditions for the functional use of these modes are not met. In bargaining mode, those demanding concessions are always countries like France and Italy. By contrast, bargaining is functional if positions are reshuffled over time. The dysfunctional state of play has not (yet) cemented into a Franco-Italian coalition^18^ mostly because of the different structural position of the two countries (only France is within the EDP) and because of the political risks of opening explicit hostilities with Germany—which would mean re-negotiating the Treaty on Stability and Growth and the architecture of the European Semester.^19^

In the Commission appointed in 2014, the new French Commissioner, Moscovici, has limited room for manoeuvre—he is subordinate to strong supporters of the ‘hierarchical’ mode. This threatens to polarize positions and create situations that are difficult to sustain politically, as shown by the domestic problems encountered by Valls and Hollande in France and their, perhaps desperate, attempt to confront Germany in early October 2014, after having suffered huge popularity and electoral losses to the advantage of the extreme right-wing Front National.

Neither did we find the conditions for functional hierarchical learning. In fact, the ‘solution’ is not known in advance, as shown by the objective difficulty of establishing among many other things the exact meaning of ‘slowdown’, ‘strong’ and ‘full’ convergence—to paraphrase Padoan’s letter to Kallas. Fundamentally, there is no shared belief on how to re-kindle growth in Europe and the precise implications of social investment are contested.

This ambiguity about growth mechanisms in the current economic conditions is exactly the reason why we find so much bargaining. The officers at DGECFIN of the Commission have no way of predicting whether the Italian government is right in making commitments about privatization and the temporary nature of the Italian deviation from the balanced budget target. These officers are in Brussels, not in Rome, and even if they were in Rome they would not be able to predict basic facts like the duration of an Italian government. All this suggests dysfunctional learning: the conditions for ‘hierarchy’ and ‘bargaining’ are not met.

Our findings show that learning-as-framework is useful to identify the prevalent mode of learning and to find out whether an organization or political system learns functionally or not. Does learning-as-framework also suggest reforms? We argue that for the EU, the solution is either to adopt other varieties of learning like reflexivity (perhaps via the ‘social investment’ debate and new shared beliefs on growth) and epistemic learning (if a new post-austerity intellectual consensus emerges one day soon), or to work on the scope conditions for functional learning in the prevailing modes of bargaining and hierarchy. More explicit mutuality in the EU would alleviate the problems with bargaining—where individual member states try to dominate. Something like this may appear from outside the European Semester, with the current activity on the Banking Union. Or, thinking of the scope condition for hierarchy, the Commission and the Council could intensify the implementation of fiscal surveillance provisions to reduce asymmetries between principal and agents, but with increasing problems of legitimacy and reduced policy autonomy at home for democratically elected governments. One way or another, the road to fiscal convergence, reforms and growth presents a set of difficult choices for the EU’s policy-makers.

## Footnotes

1 Social investment is about increasing the skills and capability of people so that they can fully participate to social life and in the labour market (Kvist 2015; Morel et al. 2014). The Commission presented the social investment strategy in February 2013 (one communication, a recommendation and eight staff papers). The Council endorsed it in October 2013, and in 2014 the Commission informed its Annual Growth Strategy with the objectives of the social investment strategy.

2 http://ec.europa.eu/europe2020/index_en.htm.

3 For a short guide to EU fiscal governance see: http://ec.europa.eu/economy_finance/articles/governance/2012-03-14_six_pack_en.htm.

4 The Treaty was signed by all EU governments bar Britain and the Czech Republic—the Czech cabinet, however, decided to join the Treaty later on, in March 2014. Croatia joined the EU in 2013 and has not signed yet.

5 Interview with *Il Fatto Quotidiano TV*; statement retrieved from the press agency www.ansa.it on 13 September 2014.

6 The famous ECB ‘confidential letter’ to Berlusconi of 5 August 2011 dictated a set or urgent reforms. It remained secret until 29 September when *Corriere della Sera* published it.

7 www.globaleconomicanalysis.blogspot.fr. Accessed on 10 May 2014.

8 Marco Galluzzo *Renzi sfida Merkel: sto con la Francia, non siamo studenti,* Corriere della Sera, 3 October 2014, p. 2.

9 Maria Teresa Meli *Renzi ha più alleati. E rilancia la politica contro i tecnocrati*, Corriere della Sera, 2 October 2014.

10 Both letters were retrieved from the official Italian government website www.mef.gov.it. Accessed on 20 April 2014.

11 Marco Zatterin, *Rinvio del pareggio, l’UE apre* La Stampa, 17 April 2014, p. 5.

12 Francesca Schianchi *Emma Bonino: Su Lady Pesc avrei fatto un’altra scelta*, La Stampa, 12 September 2014.

13 Hollande too acknowledged that Italy had done more. Speaking of policy reforms he said: “En Italie, nous le saluons et en France nous le voulons” (We welcome the reforms in Italy, we want the reforms in France). *Hollande et Renzi confrontent leurs réformes*. Le Figaro, 25 February 2015, p. 20.

14 EurActiv reported on German MEP Herbert Reul’s outrage to flexibility *à la carte*. *L’Allemange outré par la clémence de Bruxelles sur le deficit français*, 27 February 2015. www.euractiv.com. Accessed on 28 February 2015.

15 http://www.rastanews.eu/PWA_uploads/eurozone-flaws-final.pdf.

16 Carlos Yárnoz, *Hollande y Renzi presumen de liderar el giro politico de la UE*. El Pais 25 February 2015, p. 3. (Note the emphasis on ‘they presume’ in the title…“Hollande and Renzi presume to lead the political game of the EU”).

17 Présidence de la Republique: *Relevé de conclusions du XXXIIème sommet franco-italien*. Paris, 25 February 2015. “Flexibilité inscrite dans le règles” (p. 6).

18 Massimo Franco, “La crescita invocate come antidoto alla crisi” *Corriere della Sera*, 2 October 2014 argued that “the dream of a Franco-Italian axis is suggestive as well as it is controversial”.

19 The Italian economist Lucrezia Reichlin is close to suggesting to go beyond the literal interpretation of the Treaty, arguably with a modification of the underlying regulations in her editorial for the main Italian daily newspaper *La cosa giusta che non facciamo*, Corriere della Sera, 3 October 2014, pp. 1–2. On modifying the underlying regulations, see Alberto D’Aregnio, *Asse di ferro Renzi-Napolitano*, La Repubblica, 4 October 2014, pp. 6–7.

## References

[CR1] Aghion P, Gilbert C, Cohen E (2014). Changer de Modèle.

[CR2] Bauer M, Becker S (2014). The unexpected winner of the crisis: The European Commission’s role in economic governance. Journal of European Integration.

[CR3] Bennett CJ, Howlett M (1992). The lessons of learning: Reconciling theories of policy learning and policy change. Policy Sciences.

[CR4] Birchfield V (2013). A normative power Europe framework of transnational policy formulation. Journal of European Public Policy.

[CR5] Blyth M (2013). Austerity: The history of a dangerous idea.

[CR6] Borrás S, Radaelli CM, Joao-Rodrigues M, Xiarchogiannopoulou E (2014). The transformation of EU governance, the open method of coordination and economic crisis. The Eurozone crisis and the transformation of EU governance: Internal and external implications.

[CR7] Chang M (2013). Fiscal policy coordination and the future of the community method. Journal of European Integration.

[CR8] Copeland P (2015). The European Union and the ‘Social Deficit’. Representation.

[CR9] Copeland P, James S (2014). Policy windows, ambiguity and commission entrepreneurship: Explaining the re-launch of the European Union’s economic reform agenda. Journal of European Public Policy.

[CR10] Cyert RM, March J (1963). A behavioral theory of the firm.

[CR61] de la Porte C (2014). EU and social policy in times of crisis.

[CR11] de la Porte C, Heins E (2015). A new era of European integration? Governance of labour market and social policy since the sovereign debt crisis. Comparative European Politics.

[CR12] Dunlop CA, Radaelli CM (2013). Systematising policy learning: From monolith to dimensions. Political Studies.

[CR13] Dyson K (2014). States, debt, and power: ‘Saints’ and ‘sinners’ in European history and integration.

[CR14] European Commission. (2014a). *Recommendation for a council’s recommendation on France’s 2014 national reform plan*. Brussels, 2 June 2014. COM (2014) 411 Final.

[CR15] European Commission. (2014b). *Recommendation for a council’s recommendation on Italy’s 2014 national reform programme*. Brussels. 2 June 2014. COM (2014) 413 Final.

[CR16] European Commission. (2015). *Communication on making the best use of flexibility within the existing rules of the stability and growth pact*. Brussels. 13 January 2015. COM (2015) 12 Final.

[CR17] European Council. (2013). *Council conclusions*. 24–25 October 2013, Brussels. EUCO 169/13.

[CR18] Freeman R, Moran M, Rein M, Goodin RE (2006). Learning in public policy. Oxford handbook of public policy.

[CR19] Gocaj L, Meunier S (2013). Time will tell: The EFSF, the ESM, and the Euro crisis. Journal of European Integration.

[CR62] Haas PM (1992). Epistemic communities and international policy co-ordination: Introduction. International Organization.

[CR20] Hardie I, Howarth D (2013). Market-based banking and the international financial crisis.

[CR22] Heikkila T, Gerlak AK (2013). Building a conceptual approach to collective learning: Lessons for public policy scholars. Policy Studies Journal.

[CR23] Heipertz M, Verdun A (2010). Ruling Europe: The politics of the stability and growth pact.

[CR24] Hirschman AO (1970). The search for paradigms as a hindrance to understanding. World Politics.

[CR25] Howorth J (2004). Discourse, ideas and epistemic communities in European security and defence policy. West European Politics.

[CR26] Ioannou D, Leblond P, Niemann A (2015). European integration and the crisis: Practice and theory. Journal of European Public Policy.

[CR27] Jabko N (2006). Playing the market: A political strategy for uniting Europe, 1985–2005.

[CR28] Joerges C, Neyer J (1997). From intergovernmental bargaining to deliberative political processes. The constitutionalization of comitology. European Law Journal.

[CR30] Kvist J (2015). A framework for social investment strategies: Integrating generational, life course, and gender perspectives in the EU social investment strategy. Comparative European Politics.

[CR31] Lijphart A (1999). Patterns of democracy. Government forms and performance in thirty-six countries.

[CR32] Lindblom CE (1959). The science of muddling through. Public Administration.

[CR33] Majone G (1996). Regulating Europe.

[CR34] May PJ (1992). Policy learning and failure. Journal of Public Policy.

[CR35] Morel N, Palier B, Palme J (2014). Towards a social investment state? Ideas, policies and challenges.

[CR36] Moschella M, Tsingou E (2013). Great expectations, slow transformations: Incremental change in financial governance.

[CR37] Neimann A, Ioannou D (2015). European economic integration in times of crisis: A case of neo-functionalism?. Journal of European Public Policy.

[CR38] Pellizzoni L (2001). The myth of the best argument: Power, deliberation and reason. British Journal of Sociology.

[CR39] Puetter U (2012). Europe’s deliberative intergovernmentalism: The role of the council and European council in EU economic governance. Journal of European Public Policy.

[CR40] Quaglia L (2012). The “old” and “new” politics of financial services regulation in the European Union. New Political Economy.

[CR41] Radaelli CM (2008). Europeanization, policy learning, and new modes of governance. Journal of Comparative Policy Analysis.

[CR42] Risse T (2013). Arguing about arguing: A comment. Critical Policy Studies.

[CR43] Rittel HWJ, Webber MM (1973). Dilemmas in a general theory of planning. Policy Sciences.

[CR63] Sabatier PA (2007). Theories of the policy process.

[CR44] Sabel C, Zeitlin J (2008). Learning from difference: The new architecture of experimentalist governance in the European Union. European Law Journal.

[CR45] Salines M, Glöckler G, Truchlewski Z (2012). Existential crisis, incremental response: The Eurozone’s dual institutional evolution 2007–2011. Journal of European Public Policy.

[CR46] Savage JD, Verdun A (2015). Strengthening the European Commission’s budgetary and economic surveillance capacity since Greece and the euro area crisis’. Journal of European Public Policy.

[CR47] Schimmelfennig F (2014). European integration in the euro crisis: The limits of postfunctionalism. Journal of European Integration.

[CR48] Schimmelfennig F (2015). Liberal intergovernmentalism and the euro area crisis. Journal of European Public Policy.

[CR49] Schmidt VA (2013). Arguing about the eurozone crisis: A discursive institutionalist analysis. Critical Policy Studies.

[CR50] Schwarzer D (2012). The euro area crises, shifting power relations and institutional change in the European Union. Global Policy.

[CR51] Tosun J, Wetzel A,, Zapryanova G (2014). The EU in crisis: Advancing the debate. Journal of European Integration.

[CR52] Tsoukalis L (2011). The shattering of illusions—and what next?. Journal of Common Market Studies.

[CR53] van Hooren F, Kaasch A, Starke P (2014). The shock routine: Economic crisis and the nature of social policy responses. Journal of European Public Policy.

[CR54] Verdun A (1999). The role of the delors committee in the creation of EMU: An epistemic community?. Journal of European Public Policy.

[CR55] Vilpišauskas R (2013). Eurozone crisis and European integration: Functional spillover, political spillback?. Journal of European Integration.

[CR56] Zahariadis N (2008). Ambiguity and choice in European public policy. Journal of European Public Policy.

[CR57] Zeitlin, J., & Vanhercke, B. (2014). *Socializing the European Semester? Economic governance and social policy coordination in Europe 2020*. http://papers.ssrn.com/sol3/papers.cfm?abstract_id=2511031.

[CR58] Zito AR (2001). Epistemic communities, collective entrepreneurship and European integration. Journal of European Public Policy.

[CR59] Zito AR, Schout A (2009). Learning theory reconsidered: EU Integration theories and learning. Journal of European Public Policy.

[CR60] Zito AR (2001). Epistemic communities, collective entrepreneurship and European integration. Journal of European Public Policy.

